# Postoperative radiotherapy and external carotid artery diameter: is there a link to osseous free flap osteoradionecrosis risk?

**DOI:** 10.1007/s00784-026-06825-8

**Published:** 2026-03-26

**Authors:** Jakob Fenske, Elias Wollenberger, Eirini Nikolaidou, Daniel Zips, Max Heiland, Marcus Beck, Carsten Rendenbach

**Affiliations:** 1 Department of Oral and Maxillofacial Surgery, Charité – Universitätsmedizin Berlin, corporate member of Freie Universität Berlin and Humboldt-Universität zu Berlin, Augustenburger Platz 1, Berlin, 13353 Germany; 2 Department of Radiooncology and Radiotherapy, Charité – Universitätsmedizin Berlin, corporate member of Freie Universität Berlin and Humboldt-Universität zu Berlin, Augustenburger Platz 1, Berlin, 13353 Germany

**Keywords:** Vessel irradiation, External carotid artery diameter, Radiotherapy, Osseous free flaps, Microvascular free flaps, Osteoradionecrosis

## Abstract

**Objectives:**

Postoperative radiotherapy (PORT) is a cornerstone in the multimodal treatment of head and neck malignancies but may lead to osteoradionecrosis (ORN) inside healthy osseous free flaps. This study analyzes the role of external carotid artery (ECA) narrowing as a potential contributor to flap ORN, as prior evidence in this regard remains unclear.

**Materials and methods:**

This retrospective study analyzed 89 patients who received osseous free flaps and PORT between April 2017 and December 2024. ECA diameters were measured on pre- and post-radiotherapy computed tomographies. Applied radiation doses to the ECA and flaps were extracted from treatment plans. A Cox-proportional-hazard model was developed to identify free flap ORN predictors.

**Results:**

PORT led to a significant reduction in ECA diameter (-1.06 ± 1.05 mm, *p* < 0.001). No significant difference in ECA narrowing between patients with and without free flap ORN was found (*p* = 0.41). Multivariate analysis confirmed nicotine abuse (hazard ratio (HR) 6.66 [1.95;22.78], *p* = 0.003) as an independent free flap ORN predictor. Conversely, no significant association was found between ECA dose, flap dose, ECA narrowing, or plate exposure and ORN occurrence.

**Conclusions:**

Although PORT results in measurable ECA narrowing, this vascular change does not significantly influence free flap ORN risk. Future prevention strategies should instead prioritize known modifiable risk factors and further. aim to consider osseous free flaps as structures at risk during radiotherapy planning.

**Clinical relevance:**

While ECA irradiation leads to arterial narrowing, it was not identified as a predictor for free flap ORN formation.

## Introduction

Microvascular reconstructions using osseous free flaps and postoperative radiotherapy (PORT) are both fundamental parts of modern therapy concepts for malignant tumors of the head and neck. While both therapies show steadily improving outcomes due to evolving technical refinements and novel approach implementations, the combination of both yields the potential for various complications.

Besides prolonged intersegmental gap ossification and worse long-term healing outcomes of functional rehabilitation with dental implants, PORT can provoke osteoradionecrosis (ORN) in both the native mandibular or maxillary bone and the healthy autologous transplant bone, originating from the fibula, scapula, or iliac crest [1–3]. This complication has been reported to occur in 19% to 34% of irradiated free flaps, often resulting in flap loss and re-transplantation [4–6]. While optimal oncological control is still the major goal of PORT, de-escalating efforts are made to refine irradiation volumes to avoid unnecessary adverse events in healthy tissues [4, 7–9].

While ORN of the native jaws has been extensively studied, however not yet fully understood, free flap ORN is a comparably novel area of research. Previous studies from our and other groups identified an array of potential risk factors such as nicotine abuse and exposed osteosynthesis plates [6, 10]. The influence of radiation doses to osseous flap components remains a subject to debate [4, 7, 11]. Beyond radiation-induced tissue damage, surgical site infections represent the most frequent postoperative complication in patients undergoing microvascular reconstruction, particularly in previously irradiated fields. Infectious complications such as fistula formation or plate exposure have been shown to substantially impair wound healing and may act as downstream mediators contributing to ORN development [6, 12].

The influence of vascular abnormalities on radiation side-effects has been described and investigated by multiple researchers, resulting in local nutrient and oxygen deprivation, as well as promotion of inflammation [13–16]. Based on these observations, therapeutic strategies such as hyperbaric oxygen therapy (HBO) or the pentoxifylline-tocopherol-clodronate (PENTOCLO) protocol have been developed [17]. Besides that, macroscopic reductions in the diameter of the bone-supplying external carotid artery (ECA) have been described as a potential predictor for mandibular ORN formation [18]. Arterial narrowing following radiotherapy and its potential to result in an array of complications has been described for various regions, such as head and neck, pelvic or coronary vessels [19–21]. In fact, Hanubal et al. investigated the potential influence of ipsilateral ECA diameter aberrations on free flap ORN and found no significant correlations [5]. However, no separate local dose-toxicity relationship analyses on both the ECA and the free flap have been conducted to investigate whether certain doses provoke increased ECA narrowing and thus, potentially, a higher likelihood for free flap ORN formation. Moreover, ECA narrowing could also play a role in ORN pathogenesis in the combined context of further previously identified risk factors such as nicotine abuse and osteosynthesis plate exposure.

To further explore potential drivers of this relevant side-effect of multimodal therapy, we retrospectively analyzed an irradiated patient cohort, incorporating clinical and radiooncological analyses of both osseous flaps and ECAs, as well as multivariate predictive models on free flap ORN formation. With this study, we aim to assess the influence of aberrant fluid dynamics potentially caused by dose-dependent ECA narrowing and its influence on free flap ORN. Thereby we add to the current evidence on ORN formation, to prevent and address this pathology in future interdisciplinary therapy concepts.

## Materials and methods

### Hypotheses and study design

In this study, the following hypotheses were formulated: (1) PORT leads to a significant reduction of ECA diameter, (2) patients with free flap ORN show an increased ECA diameter reduction following PORT compared to those without ORN, and (3) the amount of radiation dose applied on the ECA is a relevant predictor for free flap ORN.

All patients who received PORT following resection of oral malignancies with microvascular autologous osseous free flaps (fibula free flap (FFF), scapula free flap (SFF), deep circumflex iliac artery free flap (DCIA)) reconstruction between April 2017 and December 2024 were deemed eligible for enrollment. Patients below the age of 18 years during surgery, without accessible radiotherapy treatment plans and/or without available follow-up computed tomography (CT) were excluded.

Demographic and therapy-specific parameters as well as follow-up documentations were obtained from digital patient records until February 2025. For initial analysis, planning CTs acquired postoperatively and prior to PORT were employed. To record ECA diameter progression, follow-up CTs were analyzed. Follow-up CTs had to be acquired at least 6 months after completion of PORT to account for potential acute radiation-induced soft tissue edema, possibly leading to vessel compression [18]. Free flap ORN was diagnosed in previously irradiated osseous flaps exhibiting free flap bone exposure with or without inflammation, radiological signs of osteonecrosis and histopathological confirmation. Free flap loss was defined as the need for removal of the transplanted free flap tissue due to tissue necrosis in an unlimited time frame during follow-up. To distinguish ORN from early postoperative infectious complications, only cases occurring ≥ 90 days after surgery were considered. Acute and early-onset surgical site infections were not classified as ORN events and were not included as outcome variables in this analysis. This study followed the principles of the Declaration of Helsinki and ethical approval was granted by the local ethics committee at Charité–Universitätsmedizin Berlin (EA4/109/25).

### Analysis of radiotherapy treatment plans and computed tomographies

**Fig. 1:**
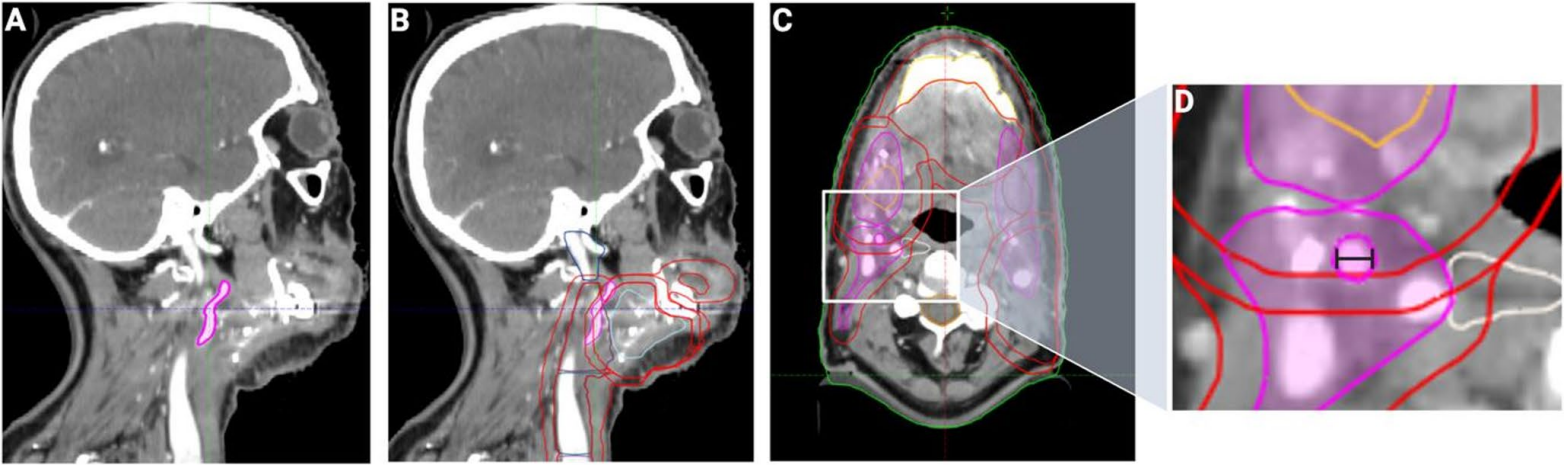
Exemplary depiction of the external carotid artery (ECA). The delineated ECA (magenta) is depicted in sagittal view of the planning computed tomography (CT) without (**A**) and with (**B**) corresponding radiation volumes. (**C**) shows the axial view of the radiation volumes with the ECA. Inset (**D**) presents an exemplary measurement of the ECA diameter (black line)

As previously described, osseous aspects of free flaps were manually contoured in the planning CT and treatment plan, to determine applied mean (Dmean) and maximum (Dmax) doses on the bone flaps [6, 11, 22]. Additionally, ECAs on the side of anastomosis (“ipsilateral”) were contoured congruently starting inferiorly at the first axial CT slice cranial to the carotid bifurcation up to the superior branching point of the maxillary and superficial temporal arteries. Applied Dmean and Dmax were automatically calculated post-hoc and acquired from the dose-volume-histograms. Maximum axial ECA diameters were measured at its largest extension at the inferior border of segmentation as described above and previously [5, 18]. Figure 1 details exemplary views of delineated and measured ECAs. All treatment plans were managed and analyzed using ARIA OIS (Version 16, Varian Medical Systems, Palo Alto, CA, USA) and previously validated by board-certified radiooncologists and medical physicists.

Congruently, ECA diameters were similarly determined on follow-up CTs. Follow-up CTs were acquired and validated by board-certified radiologists during routine oncological follow-up examinations. All ECA diameters were measured by two independent investigators (J.F. and E.W.) with knowledge of the vascular anatomy in the head and neck area using the open-source software 3D Slicer [23]. Investigators were blinded to outcome during analysis. For all measurements, CTs had a maximum slice thickness of 1.0 mm.

### Statistical analysis

Acquired data were stored and managed using spreadsheet software (Microsoft Excel 2024, Microsoft Corporation, Redmond, WA, USA). Statistical analyses were performed in RStudio for Mac (Version 2024.12.1 + 563). A p-value threshold of < 0.05 was considered statistically significant and adopted for all analyses. No missing values were present in this cohort throughout the analyzed variables. Baseline characteristics were analyzed descriptively and reported using means and standard deviations (SD) and median with interquartile range (IQR) were reported. Intraclass correlation coefficients (ICC) were calculated for analysis of interobserver agreement in diameter measurements. Qualitative variables were analyzed using Chi-squared test. Quantitative variables were assessed using Mann-Whitney U test for independent samples or Wilcoxon signed-rank test for paired samples. Subsequently, a multivariate predictive model for free flap ORN occurrence was developed via a Cox-proportional hazard model to account for different times-to-event and follow-ups. Dmean and Dmax on both ECA and osseous free flaps, absolute ECA diameter reduction as potentially relevant clinical and radiological features, as well as previously identified risk factor nicotine abuse and osteosynthesis plate exposure were integrated as covariates [6]. Hazard ratios (HR) and 95% confidence intervals (95%-CI) were calculated.

## Results

### Baseline characteristics

A total of 89 patients (*n* = 28; 32% females) with a mean age of 63.9 ± 9.2 (95%-CI [61.9;65.9]) years were included in the final analysis. Eighty-six patients (97%) underwent reconstruction of segmental mandibular defects, while three patients (3%) received osseous free flaps for maxillary reconstruction. Free flap surgery was performed using virtual preoperative planning of both the resection and the donor site via individual digitally constructed cutting and fixation guides. All patients received intravenous antibiosis for one week postoperatively using amoxicillin/clavulanic acid or clindamycin. In the case of infections in the flap area during follow-up, patients received local antiseptics and oral antibiotics. Patients received PORT via volumetric-modulated arc therapy (VMAT; *n* = 88; 99%) or tomotherapy (*n* = 1; 1%), starting 49.0 ± 24.2 (95%-CI [47.0;50.9]) days after surgery with a mean duration of 40.3 ± 5.9 (95%-CI [38.3;42.3]) days. Among free flaps, 77 (87%) patients received a FFF, 9 (10%) received a SFF and 3 (3%) received a DCIA. During free flap surgery, direct branches of the ECA were used for arterial anastomosis. Free flap ORN occurred with a frequency of 23% (*n* = 20) and a mean time to diagnosis of 19 (range 7–57) months after surgery. Only patients with the diagnosis of free flap ORN experienced free flap loss (*n* = 11; 12%). No free flaps loss occurred in patients without free flap ORN. Patients with free flap ORN had a longer follow-up period (*p* = 0.02), higher rates of nicotine (*p* = 0.001) and alcohol abuse (*p* = 0.01), flap loss (*p* < 0.001) and osteosynthesis plate exposure (*p* = 0.009). Table 1 details the baseline characteristics of the cohort.

**Table 1:** Baseline characteristics of the total cohort and the groups with and without free flap osteoradionecrosis (ORN). Values are reported as absolute and relative frequencies unless otherwise stated. The p-values are reported for group comparison between patients with and without free flap ORN

Parameter	Total Cohort (*N* = 89)	Free Flap ORN (*n* = 20)	No Free Flap ORN (*n* = 69)	*p* (ORN vs. No ORN)
Demographic Parameters				
Age [years ± SD]	63.9 ± 9.2	62.1 ± 9.0	64.4 ± 9.3	0.21
Female	23 (32%)	5 (25%)	23 (33%)	0.67
Maximum follow-up [months ± SD]	34.0 ± 21.4	44.6 ± 24.7	30.9 ± 19.5	0.02
BMI [kg/m^2^ ± SD]	23.6 ± 4.8	23.0 ± 3.8	23.8 ± 5.1	0.56
Time to follow-up CT [months ± SD]	10.1 ± 4.4	9.3 ± 2.7	10.3 ± 4.8	0.75
Comorbidities				
Nicotine abuse	41 (46%)	16 (80%)	25 (36%)	0.001
Alcohol abuse	27 (30%)	11 (55%)	16 (23%)	0.01
Arterial hypertension	33 (37%)	8 (40%)	25 (36%)	0.96
Arteriosclerosis	11 (12%)	2 (10%)	9 (13%)	> 0.99
Hyperlipidemia	7 (8%)	2 (10%)	5 (7%)	> 0.99
Disease and Therapy Specifications				
FFF	77 (87%)	17 (85%)	60 (87%)	> 0.99
SFF	9 (10%)	2 (10%)	7 (10%)	> 0.99
DCIA	3 (3%)	1 (5%)	2 (3%)	> 0.99
Mandibular defect	86 (97%)	19 (95%)	67 (97%)	> 0.99
Maxillary defect	3 (3%)	1 (5%)	2 (3%)	> 0.99
Prior head and neck radiotherapy	2 (2%)	0 (0%)	2 (3%)	> 0.99
Prior systemic chemotherapy	2 (2%)	0 (0%)	2 (3%)	> 0.99
Concomitant postoperative chemotherapy	36 (40%)	8 (40%)	28 (41%)	> 0.99
Osteosynthesis plate exposure	26 (29%)	11 (55%)	15 (22%)	0.009
Flap loss	11 (12%)	11 (55%)	0 (0%)	< 0.0001
Radiooncological Specifications				
VMAT	88 (99%)	20 (100%)	68 (99%)	> 0.99
Tomotherapy	1 (1%)	0 (0%)	1 (1%)	> 0.99
Days to PORT after surgery [days ± SD]	49.0 ± 24.2	60.4 ± 43.8	45.6 ± 13.2	0.29
PORT duration [days ± SD]	40.3 ± 5.9	41.8 ± 7.1	39.8 ± 5.5	0.23
Mean osseous free flap Dmean [Gy ± SD]	57.9 ± 4.5	59.5 ± 5.2	57.4 ± 4.3	0.11
Mean osseous free flap Dmax [Gy ± SD]	62.3 ± 4.7	63.2 ± 4.9	62.0 ± 4.6	0.48
Mean ECA Dmean [Gy ± SD]	52.4 ± 8.6	49.9 ± 10.2	53.1 ± 8.0	0.19
Mean ECA Dmax [Gy ± SD]	57.0 ± 7.4	54.6 ± 11.0	57.7 ± 6.0	0.73

*ORN*=osteoradionecrosis; *SD*=standard deviation; *BMI*=body mass index; *CT*=computed tomography; *FFF*=fibula free flap; *SFF*=scapula free flap; *DCIA*=deep circumflex iliac artery free flap; *VMAT*=volumetric-modulated arc therapy; *PORT*=postoperative radiotherapy; *Gy*=Gray; *ECA*=external carotid artery

### Influence of PORT on ECA diameter

All patients received radiation doses to the ipsilateral ECA during PORT. ECAs were exposed to a Dmean of 52.4 ± 8.6 (95%-CI [50.4;54.4]) Gy and a Dmax of 57.0 ± 7.4 (95%-CI [55.0;59.0]) Gy. Patients presented with a mean ECA diameter of 6.0 ± 1.5 mm (median (IQR) 5.8 (5.0–7.0) mm) during planning CT and 5.0 ± 1.0 mm (5.0 (4.3–5.6) mm) during follow-up CT, resulting in a mean absolute diameter reduction of.

−1.06 ± 1.28 mm (−0.9 (−1.9-0) mm) and a mean relative reduction of 15.3% ± 17.9% (16.6% (0–27.4.4%)). ICC analysis revealed good agreement between measurements among the two investigators (ICC = 0.93 [0.91;0.98] in planning CT and ICC = 0.98 [0.97;0.99] in follow-up CT).

Paired comparison of pre- and post-PORT measurements revealed a significant reduction in absolute (*p* < 0.001) ECA diameters. Spearman correlation showed no direct significant correlation between relative or absolute diameter reduction and applied ECA Dmax (absolute: *p* = 0.08, *r* = 0.19; relative: *p* = 0.22, *r* = 0.31) or Dmean (absolute: *p* = 0.39, *r* = 0.09; relative: *p* = 0.62, *r* = 0.05).

### The role of ECA diameter and PORT on free flap ORN

**Fig. 2:**
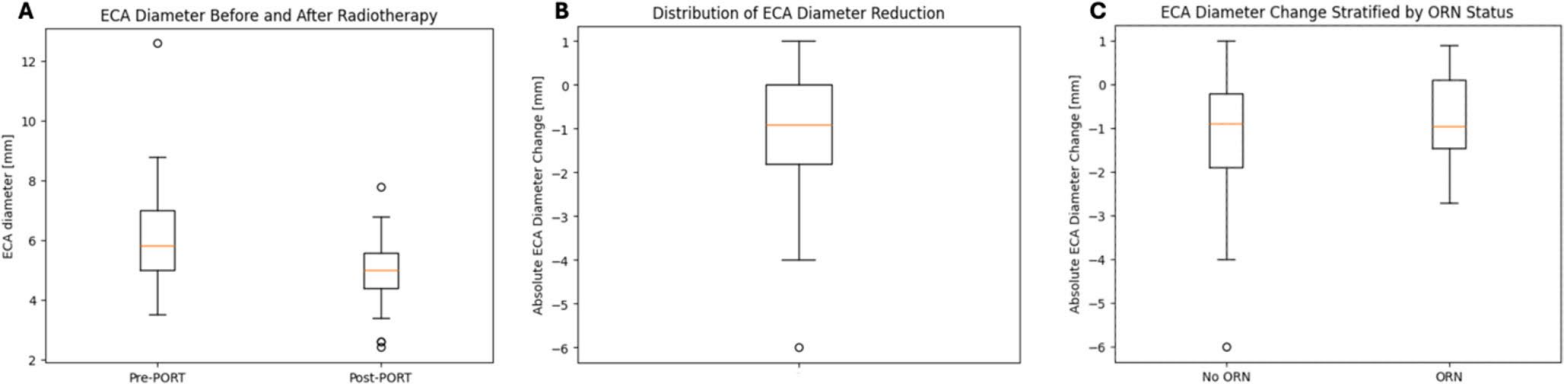
Boxplots of (**A**) external carotid artery (ECA) diameter before and after postoperative radiotherapy (PORT) in mm, (**B**) distribution of ECA diameter reduction among all patients in mm and (**C**) ECA diameter change by free flap osteoradionecrosis (ORN) status

Patients that developed free flap ORN presented with an ECA diameter reduction of −0.79 ± 1.02 mm or 11.5% ± 16.2%. Patients without ORN had an ECA diameter reduction of −1.14 ± 1.34 mm or 16.5% ± 18.3% (Fig. 2). The mean difference in absolute ECA diameter reduction between patients with and without free flap ORN was 0.36 [−0.20;0.92] mm. In group comparison, no significant differences were found in absolute (*p* = 0.41) and relative (*p* = 0.34) diameter reduction between cases with and without ORN.

The Cox-proportional hazard model revealed no significant influence of ECA Dmean (HR 0.95 [0.83;1.09], *p* = 0.46) or Dmax (HR 0.99 [0.86;1.13], *p* = 0.87), free flap Dmean (HR 1.36 [0.95;1.96], *p* = 0.09) or Dmax (HR 0.82 [0.59;1.14], *p* = 0.23), absolute ECA diameter reduction (HR 1.37 [0.92;2.06], *p* = 0.13), or osteosynthesis plate exposure (HR 2.09 [0.76;5.73], *p* = 0.15). Conversely, nicotine abuse (HR 6.66 [1.95;22.78], *p* = 0.003) was confirmed as independent predictors of free flap ORN. Table 2 presents a comprehensive overview of the logistic regression results.

**Table 2:** Results of the Cox-proportional hazard model on the risk of developing Osteoradionecrosis (ORN) inside the osseous free flap. Results are presented as hazard ratios (HR) with 95% confidence intervals (CI) and p-values (p)

Covariates		Hazar ratio (HR)	95%-CI	*p*
Radiooncological Parameters				
	ECA Dmean	0.95	[0.83;1.09]	0.46
	ECA Dmax	0.99	[0.86;1.13]	0.87
	Osseous free flap Dmean	1.36	[0.95;1.96]	0.13
	Osseous free flap Dmax	0.82	[0.59;1.14]	0.15
Clinical and Radiological Parameters				
	Absolute ECA diameter reduction	1.37	[0.92;2.06]	0.13
	Nicotine abuse	6.66	[1.95;22.78]	0.003
	Osteosynthesis plate exposure	2.09	[0.76;5.73]	0.25

*OR=*odds ratio;* 95%-CI = *95% confidence intervals;* ECA*=external carotid artery

## Discussion

As a notable morbidity-increasing side-effect of PORT, free flap ORN causes clinically relevant damage to healthy transplanted tissues. While nicotine abuse, exposed osteosynthesis plates and, potentially, increased radiation doses have been identified as free flap ORN predictors, the role of arterial narrowing has not been sufficiently investigated yet. Therefore, this study aimed to identify ECA narrowing, an arterial territory commonly used for free flap anastomosis, as a potential driver in free flap ORN cases. Although post-radiotherapy ECA narrowing was confirmed, no significant influence on ORN formation was found.

Analysis of pre- and post-radiotherapy CTs revealed a consistent significant decline of ECA diameter following radiooncological intervention. Radiation-induced carotid stenosis or ECA narrowing following irradiation of the upper aerodigestive tract have been described by various authors [24, 25]. In this study, longitudinal ECA measurements have been performed by two investigators, reaching excellent agreement according to ICC as a quality measure, comparable to other CT vascular measurement trials [26]. Our results align with the results of Hanubal et al., who found mean diameter reductions of 0.4 mm, but exceed these values with reported mean reductions of 1.06 mm in this study [5]. However, an ultrasonic-based study by Mohammadkarim et al. found common carotid artery diameter reductions of 0.86 mm, further approximating our results [27].

Besides that, correlation analysis revealed no direct dose-toxicity relationship between applied mean or maximum doses and ECA narrowing. This may be due to Dmean and Dmax values ranging between 51 and 58 Gy in this study. Although these exceed the facultative (“CAN”) recommendation of the Danish Head and Neck Cancer Group (DAHANCA) 2020 guideline for stenosis prevention, they remain at the lower end of the dose spectrum typically used for head and neck cancers, which is described as a lower risk factor for carotid toxicity by Leboucher et al. [28, 29]. In conclusion, ECA narrowing is confirmed as a relevant consequence of PORT, but linear dose-toxicity-relationships do not appear to be relevant in the lower end of the dose spectrum.

The main research question of this study was to investigate whether ECA narrowing has a predictive value for free flap ORN, based on similar findings for native jaw ORN by Ishibashi et al. [18]. In our cohort, patients with and without free flap ORN had comparable ECA diameter reductions without significant differences. This finding echoes results of Hanubal et al., who found no significant connection between ECA narrowing and free flap ORN formation [5]. In multivariate analysis, both ECA dose and narrowing were not predictive for ORN as well. However, the multivariate model confirmed nicotine abuse as a relevant factor [6]. Consequently, ECA sparing or dose reduction to cervical vessels does not seem to be necessary to prevent free flap ORN based on current evidence.

Nevertheless, a possible role of ECA branches used for anastomosis, such as the facial artery, or flap pedicle artery stenosis cannot be ruled out. While Hanubal et al. investigated anastomosis diameters and found no relevance for free flap ORN formation, this analysis was deemed unfeasible due to inconsistent and irreproducible identification of anastomoses in planning and follow-up CTs [5]. Of note, osseous free flaps often become clinically supplied by multiple local soft or hard tissue, or even contralateral in case of extensive reconstructions, arteries, further questioning the long-term relevance of a single pedicle artery. However, this remains a subject of interest and could potentially be studied using ultrasound techniques in future trials [30]. Currently, although flap pedicle delineation during PORT planning has been described, there is insufficient evidence for regular recommendation, especially regarding free flap ORN formation [8, 31].

While macroscopic vessel analyses and influences are relevant for PORT planning and understanding of broader pathophysiology, perturbed microcirculation may be a more relevant factor in deciphering free flap ORN pathogenesis. In detail, aberrant bone microcirculation has been described as a key driver for ORN in Marx’ hypoxic-hypocellular-hypovascular tissue damage theory and was later confirmed in an animal model for early ORN lesions [14, 32]. To further refine these observations and to add to current knowledge concerning the role of vascular damage in free flap ORN, high-resolution CT scans or micro-CT scans of irradiated bone specimens could be used for microcirculation analysis, as well as computational fluid dynamics finite-element modelling to gain insights into their role in ORN pathogenesis [33, 34]. In addition to radiation-induced vascular and microcirculatory changes, infectious complications must be considered when interpreting free flap ORN pathogenesis, which are common following microvascular reconstruction in irradiated patients, and may impair wound healing through chronic inflammation and unfavorable bacterial colonization [35]. Late infectious sequelae such as plate exposure or fistula formation may act as downstream mediators and precursors for ORN formation. Therefore, besides optimization of radiotherapy protocols, vigilant monitoring of late-onset infections during follow-up is necessary to reduce the rates of free flap ORN. Moreover, as previously discussed, complete sparing of or dose reduction inside osseous free flaps seems to be the most relevant aspect in PORT planning for morbidity-optimized, yet oncologically beneficial head and neck radiotherapy [6, 11].

In summary, large artery irradiation does not seem to be a key driver in free flap ORN pathogenesis. The focus for future multimodal concepts should lie on developing patient-individual, interdisciplinary therapy planning of PORT with an emphasis on considering osseous free flaps as structures at risk and minimizing applied radiation doses to these vulnerable structures.

## Limitations

This study has limitations that must be noted when interpreting the results. First, the retrospective nature of this analysis introduces inherent bias and prevents inferring causation from observation. Likewise, radiotherapy plans were contoured and analyzed post-hoc. Second, the limited sample size leads to a rather exploratory and hypothesis-generating analysis and limits generalizability. Third, all patients were treated at one hospital, leading to high internal, but potentially limited external validity, depending on surgical and radiooncological concepts. Fourth, metal CT artifacts prevented full superior identification and segmentation of ECAs in some cases. However, segmentation in the area of diameter measurement was possible throughout all CTs. Fifth, distinct measurement discrepancies between both investigators could not be ruled out. However, the ICC indicated sufficient agreement. Sixth, unavailability of PORT plans or follow-up CTs led to a drop-out of some patients, potentially leading to underreporting. Seventh, ORN patients showed a longer follow-up, potentially leading to underreporting of events in the control group. However, the control group had a sufficient follow-up, exceeding the median time to flap ORN diagnosis. Lastly, only three cases occurred in the maxilla, leading to results being mainly applicable to mandibular reconstructions.

## Conclusion

In conclusion, this study confirms that PORT leads to a significant reduction in ECA diameter. However, neither the extent of ECA narrowing nor the applied ECA radiation doses were found to significantly influence the risk of osseous free flap ORN. In contrast, nicotine abuse remains a key predictor of ORN. Therefore, efforts in radiation planning in the presence of free flaps should prioritize sparing the osseous flap over routine ECA dose reduction to mitigate ORN risk.

## Data Availability

The data used to prepare this manuscript will be available from the corresponding author upon reasonable request.
